# Nonsingular Integral Terminal Sliding Mode Control for Resonant Frequency Tracking of Electromagnetic Acoustic Transducers (EMATs) Based on Fixed-Time Strategy

**DOI:** 10.3390/mi13112005

**Published:** 2022-11-17

**Authors:** Haichao Yuan, Qi Li, Ran Peng, Chuan Wang, Peng Xu, Xinxiang Pan, Minyi Xu

**Affiliations:** 1 Dalian Key Lab of Marine Micro/Nano Energy and Self-Powered System, Dalian Maritime University, Dalian 116026, China; 2 College of Marine Engineering, Dalian Maritime University, Dalian 116026, China; 3 College of Naval Architecture and Ocean Engineering, Dalian Maritime University, Dalian 116026, China; 4 School of Electronics and Information Technology, Guangdong Ocean University, Zhanjiang 524088, China

**Keywords:** EMATs, resonant frequency tracking, fixed-time strategy, nonsingular terminal sliding mode control

## Abstract

Resonant frequency tracking control of electromagnetic acoustic transducers (EMATs) remains a challenge in terms of drifting working frequency and reduced conversion efficiency caused by working environment changes. This paper presents a fixed-time nonsingular integral terminal sliding mode (FT-NITSM) control strategy for resonant frequency tracking of EMATs to realize precise and high robustness resonant frequency tracking performance. Specifically, a FT-NITSM control method with fast convergence feature is developed and a resonant frequency tracking controller for EMATs is further designed to improve the convergence speed and tracking accuracy. Fixed time stability of the proposed frequency tracking control system is proved through Lyapunov function analysis. Moreover, numerical simulations demonstrate that the FT-NITSM control strategy can ensure precise tracking of the system’s operating frequency to its natural resonant frequency in less than 3 s with a tracking error of less than 0.01 × 10^4^ Hz. With the maximum overshoot variation between −20 and 20 and error range in −5 and 5° at the steady state, the FT-NITSM control strategy can ensure the control system impedance angle θ being consistent and eventually bounded. This study provides a toolbox for the resonant frequency tracking control and performance improvement of EMATs.

## 1. Introduction

Ultrasonic transducer is a device that detects and characterizes surface and internal defects of samples via microsystem utilizing interactions between acoustic waves and the measured materials, and has proved features of high detection sensitivity, low cost, and non-invasive detection in many fields [1,2,3,4]. Two main types of piezoelectric and electromagnetic acoustic transducers are currently in use. To ensure conversion efficiency, conventional piezoelectric ultrasonic transducers require coupling agents to achieve good contact with measured samples, making it difficult to be applied for detecting high temperature, moving, and rough surfaces [5,6]. As the electromagnetic coupling effect in conductors can excite and receive ultrasonic waves, electromagnetic acoustic transducers (EMATs) have expanded applications of ultrasonic inspection to fields of high-temperature, high-speed, and long-distance online inspection, and has received wide attention from researchers in fields such as acoustics and non-destructive testing for its non-contact, repeatability, and environmental adaptability [7,8,9,10].

Improving the output power and energy conversion efficiency of EMATs through rational design is a key issue in the EMAT research area [11,12]. The suitable working frequency of EMATs is vital for their stable operation, which not only influences their impedance, efficiency, and power output, but also affects the detection sensitivity. The output power and energy conversion efficiency of EMAT reach the maximum when it works at a resonant frequency. However, parameters of equivalent inductance and equivalent resistance may change due to the high working temperature environment and sudden changes in load, resulting in drifting resonant frequency and reduction of energy conversion efficiency [13]. Therefore, it is necessary to explore effective resonant frequency tracking methods and design frequency tracking systems for EMATs, to ensure their operation within the designed resonant frequency range and maintain high transducer efficiency.

As automatic frequency tracking of EMATs becomes increasingly demanding, traditional frequency tracking methods show problems in terms of narrow frequency range and slow tracking speed [14,15,16]. To date, many new frequency tracking methods based on fuzzy control or adaptive control strategies have been reported. Zhang et al. [17] developed a fuzzy logic method to ensure the optimal search range of the resonance frequency and designed a driving and control system, and tests results showed a feasible and reliable performance. Xu et al. [18] proposed a novel hybrid technique that involves coarse adjustment by fuzzy control and accurate adjustment by PID, which could provide quick and stable tracking of parallel resonant point frequency of the ultrasonic transducer system while maintaining decent efficiency and stability. Chang [19] presented a machine learning strategy to estimate resonance frequency for piezoelectric transducers, and compared the method with artificial neural network, support vector machine, neuro-fuzzy and mega-fuzzification, and results show that the machine learning method is convenient and effective for evaluating acoustic transducer resonant frequencies. To address reduced EMATs conversion efficiency caused by high temperature, Jia et al. [20] proposed an adaptive radial basis function neural network (RBFNN) to auto tracking EMATs resonant frequency and verified its effectiveness with numerical simulation. Wang [21] proposed the resonance frequency tracking method based on admittance circle characteristics of piezoelectric acoustic transducers and verified the short-time and high-precision tracking performance through simulation work. However, most of these methods focus on frequency tracking strategies for piezoelectric acoustic transducers and some of them are only applicable to specific systems. Therefore, control effect of the current EMATs resonant frequency tracking control method should be further improved; specifically, the tracking accuracy and robustness of the designed controller should be strengthened.

As an important variable structure nonlinear control method in the field of intelligent control, sliding mode control is featured in designing the switching hyperplane according to the system’s desired dynamic characteristics, and making the system state converge from the outside hyperplane to the switching hyperplane through sliding mode controller. Compared with existing intelligent algorithms such as adaptive control, fuzzy control, and neural network control, sliding mode control has the characteristics of fast response, robustness, and insensitivity to disturbance [22,23]. In particular, sliding mode control exhibits fast convergence speed, stable tracking performance, and superb disturbance handling capability when dealing with parameter tracking problems in nonlinear systems containing errors and fluctuations [24,25]. Therefore, the advantages of sliding mode control are well suited to the needs of EMATs resonant frequency tracking control. In the field of EMATs system application, sliding mode control has been combined with observer technology to design sliding mode observer (SMO) for improving the estimation performance model error of EMATs systems with unknown signals or uncertainties under dynamic conditions. To enhance the signal-to-noise ratio (SNR) of EMATs signals, Zhu et al. [26] designed a new unscented Kalman filter (UKF) method based on an SMO to realize the effectively measurement of the pipeline wall thickness. The SMO was introduced to establish dynamic model error estimation, which achieved better observation results. To track the changing stator current and filters out harmonics that are not part of the tracking signal to achieve static tracking of the stator current, Bao et al. [27] presented a full-order sliding mode observer (SMO) method based on synchronous frequency tracking filtering. However, as sliding mode control relies on an exact mathematical model, it cannot be perfectly applied to EMATs resonant frequency tracking control problems with multiple disturbances.

This paper has abstracted the EMATs system into a concrete dynamics model for the follow-up design of the sliding mode resonant frequency tracking controller. In order to improve the control effect of the designed sliding mode controller, the combination of finite time control and sliding mode control will further improve the convergence speed and control accuracy of the tracking controller. To solve the trajectory tracking control problem of unmanned surface vehicle in the presence of complex disturbances, Wang et al. [28,29] designed an accurate tracking control strategy to ensure that the unmanned surface vehicle can quickly and accurately track the desired trajectory by combining the finite time control and terminal sliding mode control. For microelectromechanical system gyroscope with uncertainty and external disturbance, Ren et al. [30] proposed an adaptive fuzzy finite time sliding mode control strategy by comparing it with a terminal sliding mode control method, and it showed faster convergence speed. However, the disadvantage of finite time control is that it cannot eliminate the influence of the initial state of the system on the tracking control accuracy. Meanwhile, to solve the influence of the initial state on accuracy of the tracking controller, fixed time control theory is gradually developed due to the advantage of ensuring the convergence time of the system independent of the initial state [31]. For the trajectory tracking control problem of uncertain mechanical systems, Sun et al. [32] proposed a fixed-time adaptive fuzzy sliding mode trajectory control strategy, which improves the control effect and eliminates the influence of the initial state of the mechanical system on the control accuracy. In order to solve the tracking control problem of multiple AUVs formation with disturbance, Gao et al. [33] proposed a fixed-time terminal sliding mode formation control strategy to eliminate the influence of AUV initial state on formation accuracy. However, the terminal sliding mode control strategy has singularity defects. For EMATs resonant frequency tracking control problem, the initial resonant frequency of EMATs system has a substantial impact on its tracking accuracy. Therefore, inspired by the previous research, this paper combines fixed-time control theory with nonsingular integral terminal sliding-mode control to design a frequency tracking controller, which eliminates the singularity problem of traditional terminal sliding mode controller and ensures fast global convergence and improves control performance of the proposed EMATs resonant frequency tracking control strategy. Simulation studies are implemented to investigate frequency tracking validity and merits of the proposed FT-NITSM control strategy compared with the existing used adaptive control strategy and fuzzy control strategy.

The organization of the paper is as follows: Section 2 introduces dynamic modeling of the EMATs system and some preliminaries for designing controller. Section 3 presents the design process of the resonant frequency tracking control system and establishes stability of the entire control system. Simulation results that demonstrate the effectiveness and efficiency of the proposed control strategy are presented in Section 4. Conclusions are drawn in Section 5.

## 2. Preliminaries and Problem Formulation

In this section, the controller design preliminaries work such as EMATs coil impedance matching and modeling are presented. Meanwhile, lemmas and the sliding mode control theory used in this work are also introduced.

### 2.1. Preliminaries

**Figure 1:**
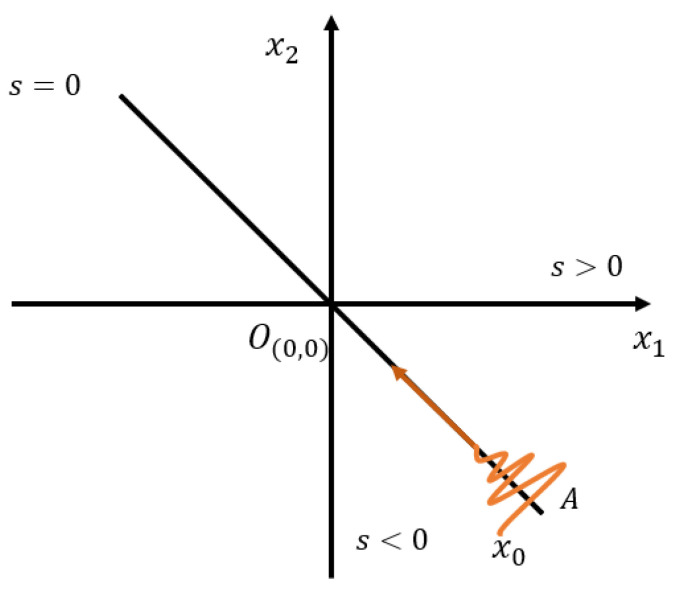
The schematic diagram of sliding mode control.

The sliding mode control is an intelligent control method based on modern control theory, which has the advantages of fast response, insensitivity to parameter changes and disturbances, no need for online system identification, and simple physical implementation. The EMATs system has internal parameter changes and external disturbances in specific working environments such as high temperature environments. The idea of sliding mode control is to pull the controlled system onto the sliding mode surface by establishing a sliding mode surface, so that the system can move along the sliding mode surface. Because the characteristics and parameters of the system only depend on the designed switching hyperplane and have nothing to do with external disturbances, sliding mode variable structure control has strong robustness. Therefore, sliding mode control can be a good approach to solve the issue of EMATs resonant frequency tracking control. Its schematic diagram is shown in Figure 1.

From Figure 1, we consider the following second-order systems:

(1) $$\begin{array}{l} {\dot{\;x}}_{1}=x_{2}\\ x_{2}=h\left(x\right)+g(x)u \end{array}$$

where, h,g are unknown nonlinear functions, for any x, we have $g(x)\geq g_{0}>0$. The purpose is to limit the motion of the system on the designed sliding surface by designing a control strategy: $s=a_{1}x_{1}+x_{2}=0$, up to the origin, that is $\underset{t\to \infty }{\lim}x\to 0$. Finally, the stability of the designed control strategy is proved by designing the Lyapunov function.

Next, lemmas that can be used in this process are shown.

**Lemma 1.**  *Consider the following nonlinear system [34]:*

(2) $$\;\;\;\;\dot{x}\left(t\right)=f\left(x\left(t\right)\right)\;x\left(0\right)=0,\;f\left(0\right)=0,t\geq 0$$

*where* $x={\left[x_{1},x_{2},\ldots ,x_{n}\right]}^{T}$*is the system state vector and* f(x(t))*is a nonlinear function defined in the neighborhood of the origin. If the system (2) is asymptotically stable and has a negative homogeneity degree, the system is finite-time stable.*

**Lemma 2.**  *Consider the following scalar system [35]:*

(3) $$\dot{y}=-\gamma_{1}y^{2-p/q}-\gamma_{2}y^{p/q},y\left(0\right)=y_{0}$$

*where* $\gamma_{1},\gamma_{2}>0$, p,q*are both positive odd integers satisfying*p<q. *System (19) is fixed-time stable and the upper bound of the convergence time satisfies:*

(4) $$T_{max}\left(y_{0}\right)=\frac{q\pi }{2\sqrt{\gamma_{1}\gamma_{2}}\left(q-p\right)}$$

**Lemma 3.**  *Consider the continuous radial bounded function V:*$R^{n}\to R_{+}\cup \left\{0\right\}$, which satisfies the following two conditions [31]:

(1) V(x)=0⇔x=0.

(2) *For all* x(t) *satisfies* $\dot{V}\left(x\right)\leq -c_{1}V^{d_{1}}\left(x\right)-c_{2}V^{d_{2}}\left(x\right)$, *where* $c_{1},c_{2},d_{1},d_{2}$ *are normal numbers, and* $0<d_{1}<1$, $d_{2}>1$. *Then, the system can achieve rapid convergence in a fixed time, and the maximum convergence time is calculated as follows:*

(5) $$T<=T_{max,2}=\frac{1}{c_{1}\left(1-d_{1}\right)}+\frac{1}{c_{2}\left(d_{2}-1\right)}$$

### 2.2. Impedance Matching of EMATs Coil

**Figure 2:**
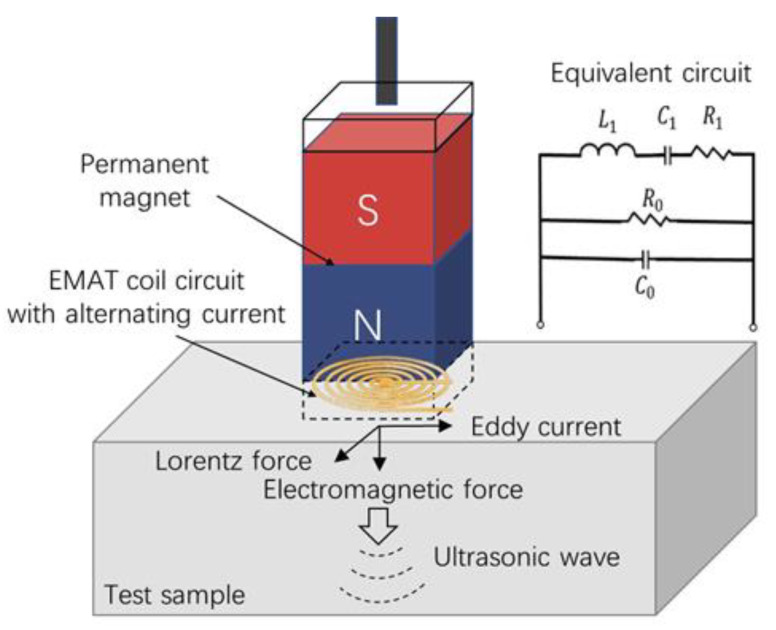
The configuration and equivalent circuit of an EMAT.

EMATs generate ultrasonic waves by utilizing electrodynamic processes in conductive metals. The exciting coil with alternating current is positioned above the conducting metal test sample. The alternating magnetic field generated by the coil acts on the conductive metal and induces an eddy current, which is under the constant magnetic field. The electrically charged mass is displaced by the force while flowing in the magnetic field, thus exciting ultrasonic waves with the same frequency as that of the incoming alternating current. The probe of EMATs is composed of a coil and a magnet and optimizing the performance of EMATs is achieved by appropriately adjusting the probe structure and parameters. A spiral coil and a vertical form of static magnetic field design are illustrated as the probe for EMATs in this work. An EMAT system operating near resonant frequency can be abstracted and equivalent to a circuit model as shown in Figure 2, in which *L*_1_ is equivalent inductance, R_1_ is equivalent resistance, *C*_1_ is equivalent distributed capacitance, *R*_0_ is dielectric loss resistance, *C*_0_ is static capacitance.

Based on the above EMAT equivalent circuit, the impedance angle θ can be expressed as follows:

(6) $$\theta =\arctan\left[\frac{\left({2L}_{1}C_{0}{+L}_{1}C_{1}-C_{0}C_{1}R_{1}^{2}\right)\omega }{C_{1}R_{1}}-\frac{L_{1}^{2}\omega^{3}C_{0}}{R_{1}}-\frac{C_{0}{+C}_{1}}{C_{1}^{2}R_{1}\omega }\right]$$

where ω represents angular frequency. The presence of static capacitance *C*_0_ causes the transducer to be capacitive and there will be a large reactive power loss, which greatly reduces the transducer power and efficiency. Therefore, this paper adopts the series inductor-parallel capacitor method shown in the above circuit to match EMATs coils. The total admittance $Y_{s}$ after matching is as follows:

(7) $$\begin{array}{l} Y_{s}{=G}_{s}{+B}_{s}j\\ =\left[\frac{1}{R_{0}}+\frac{R_{1}\omega^{2}C_{1}^{2}}{{\left({1-\omega }^{2}L_{1}C_{1}\right)}^{2}{+R}_{1}^{2}\omega^{2}C_{1}^{2}}\right]+\left[\left(C_{m}\omega -\frac{1}{{\omega L}_{s}}\right)+\frac{\left(1-\omega^{2}L_{1}C_{1}\right){\omega C}_{1}}{{\left(1-\omega^{2}L_{1}C_{1}\right)}^{2}{+R}_{1}^{2}\omega^{2}C_{1}^{2}}\right]j \end{array}$$

where $G_{s}$ is conductance and $B_{s}$ is susceptance. We equivalent the static capacitance *C*_0_ and matching capacitance *C_s_* to a new capacitance *C_m_, C_m_ = C*_0_ + *C_s_*. When $C_{m}\omega -\frac{1}{{\omega L}_{s}}=0$, we can get:

(8) $$\{\begin{array}{c} G_{s}=\left[\frac{1}{R_{0}}+\frac{R_{1}\omega^{2}C_{1}^{2}}{{\left(1-\omega^{2}L_{1}C_{1}\right)}^{2}{+R}_{1}^{2}\omega^{2}C_{1}^{2}}\right]\\ B_{s}=\left[\left(C_{m}\omega -\frac{1}{{\omega L}_{s}}\right)+\frac{\left({1-\omega }^{2}L_{1}C_{1}\right){\omega C}_{1}}{{\left(1-\omega^{2}L_{1}C_{1}\right)}^{2}{+R}_{1}^{2}\omega^{2}C_{1}^{2}}\right] \end{array}$$

Impedance angle θ is calculated as follows:

(9) $$\theta =\arctan\left[\frac{L_{1}}{R_{1}}-\frac{1}{C_{1}R_{1}\omega }\right]$$

Letting θ=0 gives:

(10) $$f_{r}=\frac{1}{2\pi \sqrt{L_{1}C_{1}}}$$

At this time, the mechanical resonant frequency $f_{s}\;$, the maximum admittance corresponding to frequency $f_{m}$ and zero admittance corresponding to frequency $f_{r}$ are equal, then:

(11) $$f_{r}=f_{s}=f_{m}=\frac{1}{2\pi \sqrt{L_{1}C_{1}}}$$

when the initial moment frequency $f=f_{0}$ is selected, we can have:

(12) $$L_{s}=\frac{1}{4\pi^{2}f_{0}^{2}C_{m}}$$

As can be noted from Equation (12), the ideal matching effect can be reached by selecting appropriate $C_{s}$ and $L_{s}$ to achieve accurate resonant frequency tracking.

### 2.3. Problem Formulation

The admittance equation of the equivalent circuit of EMATs coil after impedance matching is shown as follows:

(13) $$\begin{array}{l} Y_{s}=G_{s}+B_{s}j\\ \;=\frac{R_{1}\omega^{2}C_{1}^{2}}{{\left(1-\omega^{2}L_{1}C_{1}\right)}^{2}+R_{1}^{2}\omega^{2}C_{1}^{2}}+\left[\left(C_{m}\omega -\frac{1}{\omega L_{s}}\right)+\frac{\left(1-\omega^{2}L_{1}C_{1}\right)\omega C_{1}}{{\left(1-\omega^{2}L_{1}C_{1}\right)}^{2}+R_{1}^{2}\omega^{2}C_{1}^{2}}\right]j \end{array}$$

where:

(14) $$C_{m}=C_{0}+C_{s}$$

The impedance angle θ is calculated as follows:

(15) $$\begin{array}{l} \theta =\arctan\left(-\frac{B_{s}}{G_{s}}\right)\\ \;=\arctan[\frac{L_{1}^{2}\omega^{3}C_{m}{+L}_{1}\omega }{R_{1}}+\frac{{2L}_{1}C_{m}\omega }{C_{1}R_{1}}-\frac{{2L}_{1}}{C_{1}R_{1}L_{s}\omega }+\frac{R_{1}}{L_{s}\omega }+\frac{1}{C_{1}R_{1}\omega }-\frac{L_{1}^{2}\omega }{R_{1}L_{s}}+\frac{C_{m}}{C_{1}^{2}R_{1}\omega }-\frac{1}{C_{1}^{2}R_{1}L_{s}\omega^{3}}-C_{m}R_{1}\omega ] \end{array}$$

Let $\theta =f\left(\omega ,L_{1},R_{1},C_{1}\right)$. We assume that the equivalent inductance $L_{1}$ and resistance $R_{1}$ of the coil are smooth continuous functions of temperature and lift-off (the equivalent distributed capacitance $C_{1}$ is defined as a constant with small values and no significant changes), the impedance angle θ is defined as follows:

(16) $${\theta =f(\omega ,\;L}_{1}\left(T,\;h\right),\;R_{1}\left(T,\;h\right),{\;C}_{1})\;=F(\omega ,\;T,\;h)$$

where *T* is the ambient temperature, h is the lift-off distance, and ω, *T*, *h* are the function vectors that change continuously with time *t*: Z=[ω,T,h]. The derivative of time *t* gives:

(17) $$\dot{\theta }\left(t\right)=\frac{\partial F}{\partial \omega }\dot{\omega }+\frac{\partial F}{\partial T}\dot{T}+\frac{\partial F}{\partial h}\dot{h}$$

The design control input ω is generated by a low-pass filter, which is driven by input ν, as shown below:

(18) $$\dot{\omega }=-a\omega +v$$

where $a\in R_{+}$ is the design parameter. Then, the above formula is rewritten as follows:

(19) $$\dot{\theta }\left(t\right)=\frac{\partial F}{\partial \omega }\dot{\omega }+\frac{\partial F}{\partial T}\dot{T}+\frac{\partial F}{\partial h}\dot{h}\;\;\;\;\;\;\;\;\;=\frac{\partial F}{\partial \omega }\left(-a\omega +v\right)+\frac{\partial F}{\partial T}\dot{T}+\frac{\partial F}{\partial h}\dot{h}\;\;\;\;\;\;\;\;\;=g\left(\omega \right)v+H\left(\omega ,T,h\right)$$

where:

(20) $$g\left(\omega \right)=\frac{\partial F}{\partial \omega }v\;H\left(\omega ,T,h\right)=-a\frac{\partial F}{\partial \omega }\omega +\frac{\partial F}{\partial T}\dot{T}+\frac{\partial F}{\partial h}\dot{h}$$

In terms of above analysis, the EMATs system can be abstracted as a second-order nonlinear system, whose mathematical model can be expressed by:

(21) $$\{\begin{array}{l} \dot{\omega }=-a\omega +v\\ \dot{\theta }=g\left(\omega \right)v+H\left(z\right) \end{array}$$

## 3. Design of Proposed Controller

**Figure 3:**
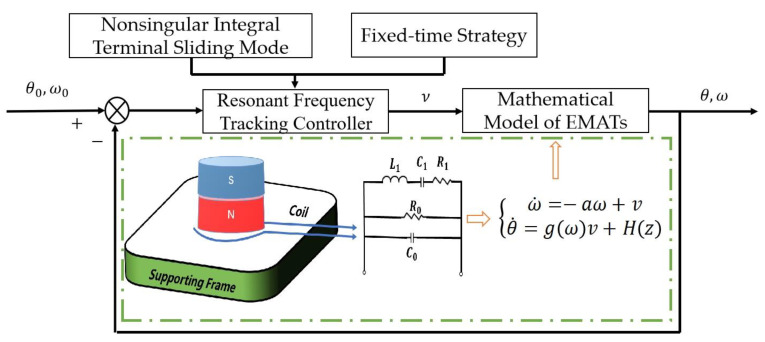
The control block diagram of FT-NITSM tracking control strategy for EMATs resonant frequency.

Combined with the research content of this paper, after the mathematical model of the controlled object EMATs is determined, combined with the sliding mode controller and the fixed time theory, the simplified control block diagram of the FT-NITSM control method for tracking the resonant frequency of the EMATs is shown in Figure 3 as follows:

To facilitate the design of the resonant frequency tracking controller later, let $x_{1}=\theta ,x_{2}=\omega$, simplify Equation (16) as follows:

(22) $$\{\begin{array}{c} {\dot{x}}_{1}=g\left(x_{2}\right)v+H\left(x_{2},T,h\right)\\ {\dot{x}}_{2}=-ax_{2}+v \end{array}$$

Define the desired tracking frequency as follows:

(23) $$\{\begin{array}{l} {\dot{\theta }}_{d}=g\left(\omega_{d}\right)v+H\left(\omega_{d},T,h\right)\\ {\dot{\omega }}_{d}=-a\omega_{d}+v \end{array}$$

From Equations (22) and (23), the tracking error is defined as follows:

(24) $$\{\begin{array}{l} \Delta_{1}=\theta -\theta_{d}\\ \Delta_{2}=\omega -\omega_{d} \end{array}$$

The derivation is as follows:

(25) $$\{\begin{array}{c} {\;\dot{\Delta }}_{1}=\dot{\theta }-{\dot{\theta }}_{d}=\left[g\left(\omega \right)-g\left(\omega_{d}\right)\right]v+\left[H\left(z\right)-H\left(z_{\omega_{d}}\right)\right]\\ {\;\dot{\Delta }}_{2}=\dot{\omega }-{\dot{\omega }}_{d}=-a\omega +v+a\omega_{d}-v_{d}=a\left(\omega_{d}-\omega \right)+\left(v-v_{d}\right) \end{array}$$

The NITSM with fixed time convergence is designed as follows:

(26) $$s=\Delta_{1}+\int_{0}^{t}\left[a_{1}sig^{b_{1}}\left(\Delta_{1}\right)+a_{2}sig^{b_{2}}\left(\Delta_{1}\right)+a_{3}sig^{b_{3}}\left(\Delta_{2}\right)+a_{3}sig^{b_{3}}\left(\Delta_{2}\right)\right]dv$$

where $a_{1},a_{2},a_{3},a_{4}$ are positive odd numbers, $b_{1},b_{2},b_{3},b_{4}$ are positive real numbers and satisfy $b_{1}=\frac{b_{3}}{2-b_{3}}$, $b_{2}=\frac{b_{4}}{2-b_{4}}$, $1<b_{3}<2$, $0<b_{4}<1$.

The derivative of the sliding surface is as follows:

(27) $$\dot{s}={\dot{\Delta }}_{1}+a_{1}sig^{b_{1}}\left(\Delta_{1}\right)+a_{2}sig^{b_{2}}\left(\Delta_{1}\right)+a_{3}sig^{b_{3}}\left(\Delta_{2}\right)+a_{4}sig^{b_{4}}\left(\Delta_{2}\right)$$

Let $\dot{s}=-\lambda_{0}s-\lambda_{1}s^{2-\frac{m}{n}}-\lambda_{2}s^{\frac{m}{n}}$. Where $\lambda_{0},\;\lambda_{1},\lambda_{2}$ indicate controller parameters, m,n represent positive odd numbers and satisfy m<n.

Then, we have:

(28) $$\left[g\left(\omega \right)-g\left(\omega_{d}\right)\right]v+\left[H\left(z\right)-H\left(z_{\omega_{d}}\right)\right]+a_{1}sig^{b_{1}}\left(\Delta_{1}\right)+a_{2}sig^{b_{2}}\left(\Delta_{1}\right)+a_{3}sig^{b_{3}}\left(\Delta_{2}\right)+a_{4}sig^{b_{4}}\left(\Delta_{2}\right)\;=-\lambda_{0}s-\lambda_{1}s^{2-\frac{m}{n}}-\lambda_{2}s^{\frac{m}{n}}$$

The resonant frequency tracking control strategy of EMATs under high temperature is designed as follows:

(29) $$\begin{array}{l} v=\frac{-\lambda_{0}s-\lambda_{1}s^{2-\frac{m}{n}}-\lambda_{2}s^{\frac{m}{n}}-\left[a_{1}sig^{b_{1}}\left(\Delta_{1}\right)+a_{2}sig^{b_{2}}\left(\Delta_{1}\right)+a_{3}sig^{b_{3}}\left(\Delta_{2}\right)+a_{4}sig^{b_{4}}\left(\Delta_{2}\right)\right]-\left[H\left(z\right)-H\left(z_{\omega_{d}}\right)\right]}{g\left(\omega \right)-g\left(\omega_{d}\right)}\\ \;=\frac{\lambda_{0}s+\lambda_{1}s^{2-\frac{m}{n}}+\lambda_{2}s^{\frac{m}{n}}+\left[a_{1}sig^{b_{1}}\left(\Delta_{1}\right)+a_{2}sig^{b_{2}}\left(\Delta_{1}\right)+a_{3}sig^{b_{3}}\left(\Delta_{2}\right)+a_{4}sig^{b_{4}}\left(\Delta_{2}\right)\right]+\left[H\left(z\right)-H\left(z_{\omega_{d}}\right)\right]}{g\left(\omega_{d}\right)-g\left(\omega \right)}\;\\ \;\;=\frac{M+N+\left[H\left(z\right)-H\left(z_{\omega_{d}}\right)\right]}{g\left(\omega_{d}\right)-g\left(\omega \right)} \end{array}$$

where $a_{1}sig^{b_{1}}\left(\Delta_{1}\right)+a_{2}sig^{b_{2}}\left(\Delta_{1}\right)+a_{3}sig^{b_{3}}\left(\Delta_{2}\right)+a_{4}sig^{b_{4}}\left(\Delta_{2}\right)=N$. $\lambda_{0}s+\lambda_{1}s^{2-\frac{m}{n}}+\lambda_{2}s^{\frac{m}{n}}=M$.

We select the following Lyapunov functions:

(30) $$V=\frac{1}{2}s^{T}s$$

The derivation is as follows:

(31) $$\begin{array}{ll} \dot{\;\;\;\;\;\;\;\;\;\;\;\;\;\;\;\;\;\;\;\;\;\;\;\dot{V}} & =s^{T}\dot{s}\\ & =s^{T}\left[{\dot{\Delta }}_{1}+a_{1}sig^{b_{1}}\left(\Delta_{1}\right)+a_{2}sig^{b_{2}}\left(\Delta_{1}\right)+a_{3}sig^{b_{3}}\left(\Delta_{2}\right)+a_{4}sig^{b_{4}}\left(\Delta_{2}\right)\right] \end{array}$$

Combining Equations (27) and (29), we can get:

(32) $$\dot{V}=s^{T}\left[\left[g\left(\omega \right)-g\left(\omega_{d}\right)\right]v+H\left(z\right)-H\left(z_{\omega_{d}}\right)+a_{1}sig^{b_{1}}\left(\Delta_{1}\right)+a_{2}sig^{b_{2}}\left(\Delta_{1}\right)+a_{3}sig^{b_{3}}\left(\Delta_{2}\right)+a_{4}sig^{b_{4}}\left(\Delta_{2}\right)\right]\;=s^{T}\left\{\left[g\left(\omega \right)-g\left(\omega_{d}\right)\right]\frac{N+M+H\left(z\right)-H\left(z_{\omega_{d}}\right)}{g\left(\omega_{d}\right)-g\left(\omega \right)}+H\left(z\right)-H\left(z_{\omega_{d}}\right)+N\right\}$$

To simplify the above Equation (32), we can get:

(33) $$\begin{array}{ll} \dot{\;\;\;\;\;\;V} & =s^{T}\left\{\left[g\left(\omega \right)-g\left(\omega_{d}\right)\right]\frac{N+M+H\left(z\right)-H\left(z_{\omega_{d}}\right)}{g\left(\omega_{d}\right)-g\left(\omega \right)}\;+H\left(z\right)-H\left(z_{\omega_{d}}\right)+N\right\}\\ & =s^{T}\left\{-\left[N+M+H\left(z\right)-H\left(z_{\omega_{d}}\right)\right]+H\left(z\right)-H\left(z_{\omega_{d}}\right)+N\right\}\\ & =-s^{T}M\\ & =-s^{T}\left(\lambda_{0}s+\lambda_{1}s^{2-\frac{m}{n}}+\lambda_{2}s^{\frac{m}{n}}\right)\\ & =-\lambda_{0}s^{2}-\lambda_{1}s^{\frac{3n-m}{n}}-\lambda_{2}s^{\frac{m+n}{n}}\\ & =-\lambda_{0}s^{2}-\lambda_{1}{\left(\frac{1}{2}s^{2}\right)}^{\frac{3n-m}{2n}}-\lambda_{2}{\left(\frac{1}{2}s^{2}\right)}^{\frac{m+n}{2n}}\\ & \leq -\lambda_{1}V^{\frac{3n-m}{2n}}-\lambda_{2}V^{\frac{m+n}{2n}} \end{array}$$

According to Lemma 2, the system will converge in a fixed time and the upper limit of the convergence time is:

(34) $$T_{s}=\frac{2n\pi }{2\sqrt{\lambda_{1}2^{\frac{3n-m}{2n}}\lambda_{2}2^{\frac{m+n}{2n}}\left(n-m\right)}}$$

It can be seen from the above that when reaching the sliding surface, $s=0,\;\dot{s}=0.$ Combining with Equation (27), we can get:

(35) $${\dot{\Delta }}_{1}=-a_{1}{\mathrm{sig}}^{b_{1}}\left({\dot{\Delta }}_{1}\right)-a_{2}{\mathrm{sig}}^{b_{2}}\left({\dot{\Delta }}_{1}\right)-a_{3}{\mathrm{sig}}^{b_{3}}\left({\dot{\Delta }}_{2}\right)-a_{4}{\mathrm{sig}}^{b_{4}}\left({\dot{\Delta }}_{2}\right)$$

According to Lemma 3, the tracking error dynamics can converge to zero along the sliding mode surface in a fixed time after the designed FT-NITSM reaches the sliding mode surface.

To sum up, the designed FT-NITSM resonant frequency tracking control strategy can achieve fixed time rapid convergence and guarantee in the approach phase and sliding phase, and the designed FT-NITSM tracking control strategy can ensure the desired resonant frequency set on the fast and stable tracking of the intrinsic resonant frequency.

## 4. Simulation and Discussion

In this section, simulations are conducted to verify the overall effectiveness of the resonant frequency tracking and control strategy of EMATs based on FT-NITSM. Specifically, in order to ensure the comprehensiveness of the simulation part and the integrity of the structure, we conducted simulation experiments and discussions.

First, fixed time control is the basis to designing the FT-NITSM tracking control strategy. In order to prove the effectiveness of fixed time control strategy, we designed and conducted comparison experiments of the traditional linear controller, finite time controller, and fixed time controller. Specific comparison processes are shown as follows:

Consider a simple system: $\dot{x}=u$

Compare the following three controllers:

$$\{\begin{array}{l} \mathrm{Traditional}\;\mathrm{linear}\;\mathrm{controller}:u=-kx\\ \mathrm{Finite}\;\mathrm{time}\;\mathrm{controller}:u=-k\times sign(x)|x{|}^{\alpha },k>0,0<\alpha <1\\ \mathrm{Fixed}\;\mathrm{time}\;\mathrm{controller}:u=-k\times sign(x)|x{|}^{\alpha }-k\times sign(x)|x{|}^{\beta },k>0,0<\alpha <1,\beta >1 \end{array}$$

**Figure 4:**
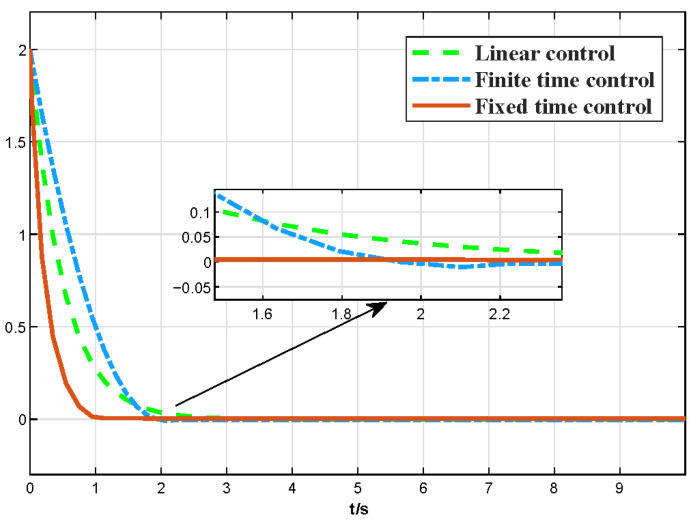
The comparative curve of fixed-time, finite-time, and linear control.

The values are as follows: k=2,β=1.5,α=0.5. The response curve of three different controllers is shown in Figure 4:

As shown in Figure 4, for the three different control strategy-based controllers, the traditional liner controller shows a very slow convergence speed although the convergence process is smooth, while the finite time controller effectively improves the convergence speed. Simulation comparison results show that the fixed-time controller exhibits faster speed and smoother performance during the whole convergence process, which demonstrates the superior performance of the fixed-time control strategy designed in this paper.

**Figure 5:**
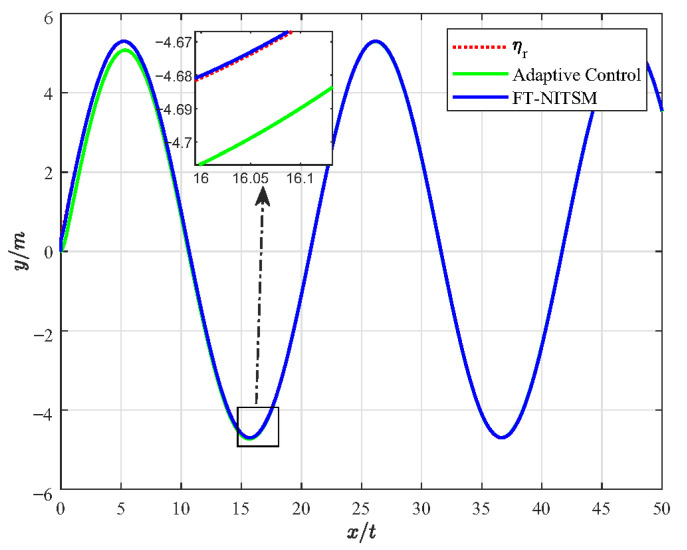
Comparison of resonant frequency tracking performance of FT-NITSM control and adaptive tracking control.

**Figure 6:**
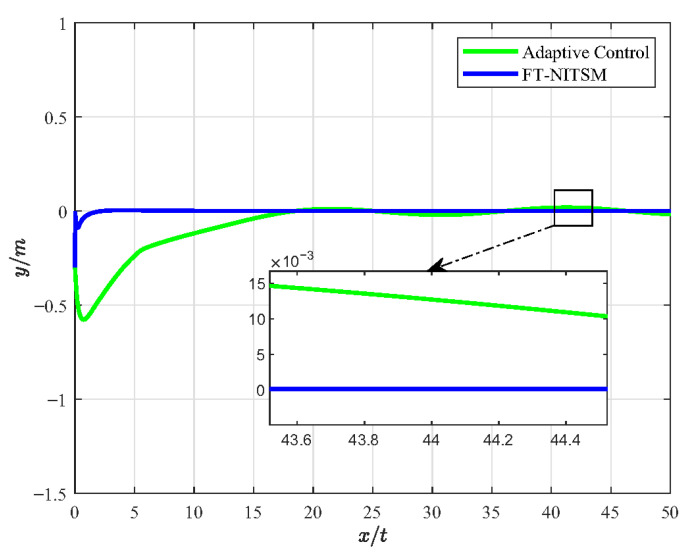
The error diagram of FT-NITSM tracking control and adaptive control.

**Figure 7:**
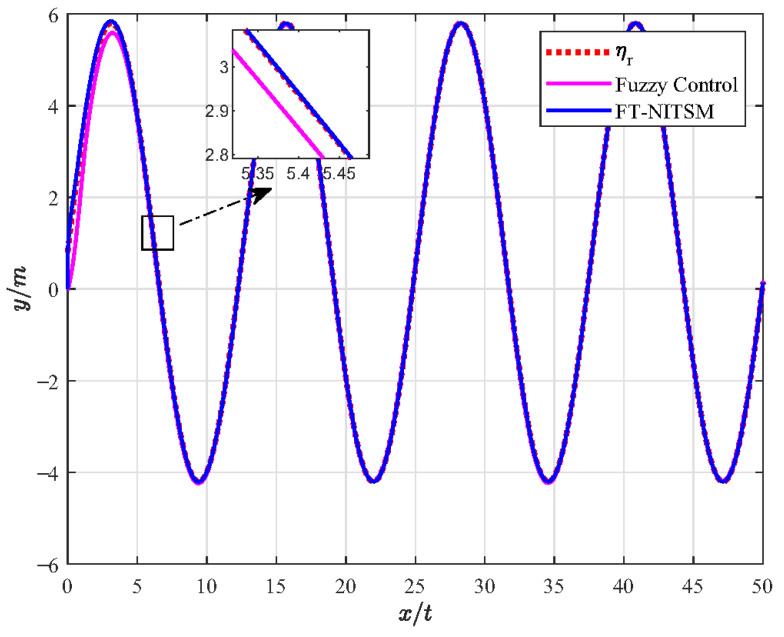
Comparison of resonant frequency tracking performance of FT-NITSM and fuzzy control.

**Figure 8:**
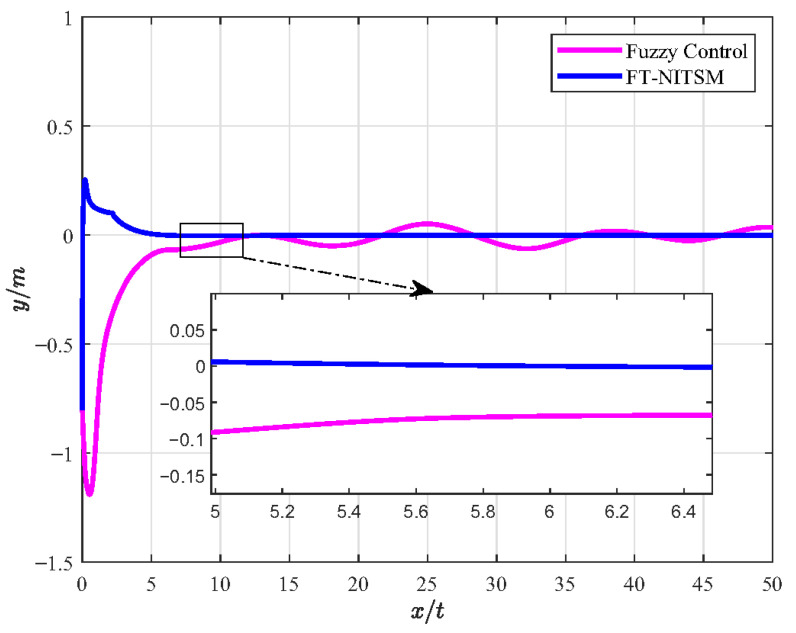
Comparison of error diagram of FT-NITSM tracking control and fuzzy control.

Furthermore, the FT-NITSM control method is the theoretical basis to designing the EMATs resonant frequency tracking control strategy. In order to prove the effective-ness of the FT-NITSM tracking control strategy, we compared it with the adaptive control strategy and the fuzzy control strategy, and designed the simulation comparison experiment as shown in Figure 5, Figure 6, Figure 7 and Figure 8.

Figure 5 presents the simulation experiment diagram of comparative analysis on resonant frequency tracking performance between the FT-NITSM control and the adaptive control. We set the expected path function as y=5sin(0.5×x)+0.8 and the simulation time as 50 s. As can be seen from the simulation results in Figure 5, the tracking speed of FT-NITSM control strategy proposed in this paper is extremely fast, and the tracking accuracy is far higher than the adaptive tracking control strategy after reaching the steady state. To further compare the tracking performance of the two methods, Figure 6 shows the comparison effect of tracking errors under the two tracking control methods, in which the tracking error of the FT-NITSM tracking control strategy designed in this paper is very small, while the maximum tracking error of adaptive tracking control strategy after tracking on the expected trajectory is greater than 0.015 m.

Figure 7 shows the simulation experiment diagram of comparative analysis between the FT-NITSM tracking control strategy and the fuzzy control strategy. We set the expected path function as y=5sin(0.3×x)+0.3 and the simulation time as 50 s. From the simulation results, it can be seen that the tracking speed and tracking accuracy of the proposed FT-NITSM tracking control strategy are much higher than those of the fuzzy control strategy. To further compare the tracking performance under the two methods, Figure 8 shows the comparison effect of tracking error under FT-NITSM tracking control strategy and fuzzy control strategy. It can be seen from Figure 8 that the tracking error of the FT-NITSM tracking control strategy designed in this paper is very small, while the maximum tracking error of the fuzzy control strategy after tracking on the expected trajectory is greater than 0.1 m. The results show that the FT-NITSM proposed in this paper has the best control effect among the three control strategies, while the fuzzy control exhibits the worst control effect. Simulation results show the effectiveness and efficiency of the tracking control strategy designed in this paper.

**Figure 9:**
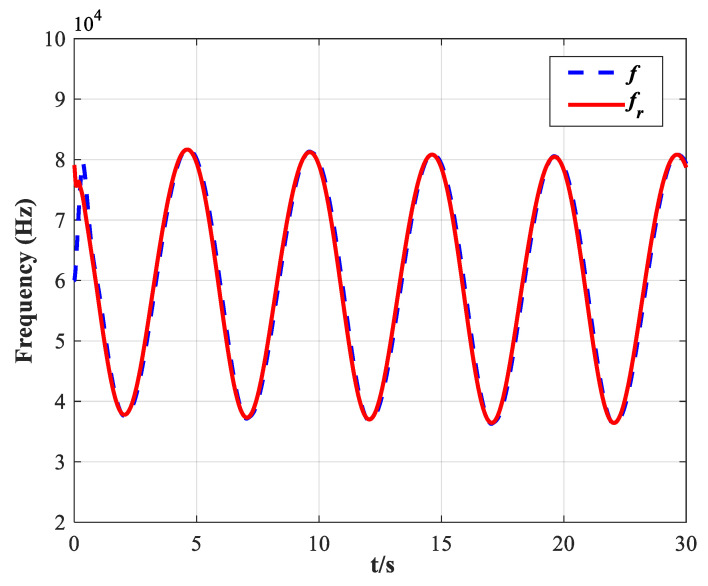
Tracking performance of the designed FT-NITSM resonant frequency tracking controller on its natural resonant frequency $f_{r}$.

**Figure 10:**
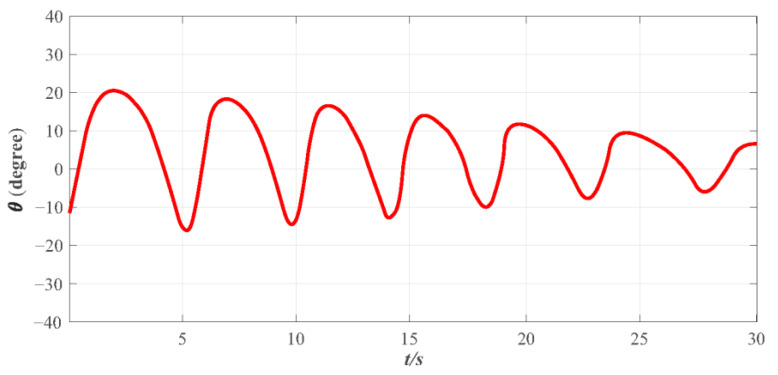
The variation of impedance angle θ under the designed FT-NITSM resonant frequency tracking controller.

**Figure 11:**
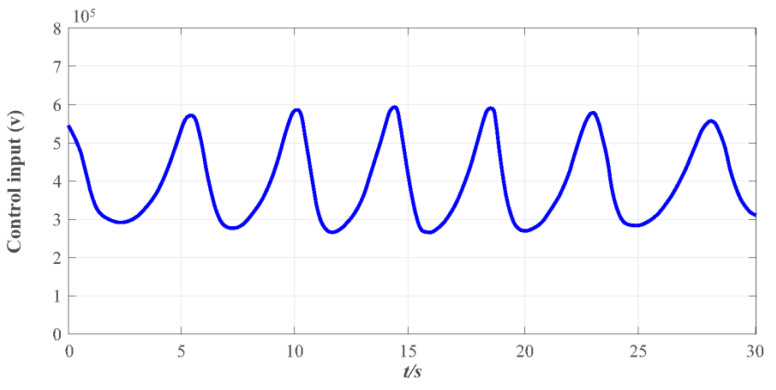
The control input curve under the designed FT-NITSM resonant frequency tracking controller.

**Figure 12:**
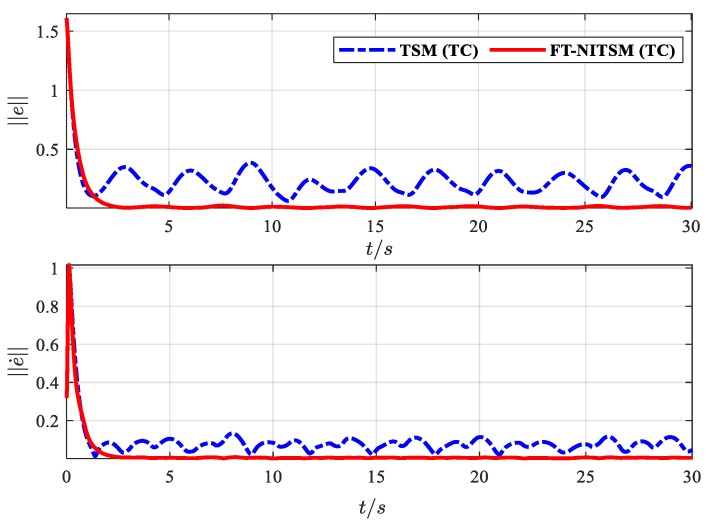
The tracking error norm comparison effect diagram of TSM tracking controller and FT-NITSM tracking controller.

Finally, to further verify that the proposed FT-NITSM control method can effectively solve the problem of EMATs resonant frequency tracking control based on the EMATs mathematical model proposed in this paper, an EMATs resonant frequency tracking simulation platform was built with Matlab/Simulink. The EMAT system starts running after the controller parameters, the dynamic performance indicators, and the initial parameters of the system have been set. Simulation results are shown in Figure 9, Figure 10, Figure 11 and Figure 12. Detailed controller parameters were selected as shown in Table 1:

Figure 9 depicts the tracking effect of the actual working frequency on the system natural resonance frequency, in which the solid line represents the natural resonance frequency $f_{r}$ of the system under the set EMATs model, while the dotted line represents the actual working frequency f of the EMATs system. The simulation time is set to be 0–100 s while the initial frequency of the system is set to be $f\left(0\right)=6\times 10^{4}$ Hz. As shown in Figure 9, the system working frequency has accurately tracked the natural resonant frequency in less than 3 s with the positive and negative tracking error is less than $0.01\times 10^{4}$ Hz, which demonstrates that the FT-NITSM tracking control strategy designed in this paper can be applied to the EMATs precise resonant frequency tracking.

In addition to fast-tracking performance to guarantee the system can work near the resonant frequency, the performance of maintaining the system to be steady and anti-interference is also vital, indicating that the system can work near the resonant frequency, and overshoot of the system impedance angle θ and the steady-state error is within a reasonable range. Figure 10 presents the effect diagram of impedance angle change in the EMATs system, and results show that the impedance angle θ of the system is consistent and ultimately bounded. The overshoot range of the system impedance angle θ change in the entire simulation stage is between −20 and 20 and the convergence speed is fast. The impedance angle maintains stability in 30 s, and the change range is stable between −5 and 5°, thus ensuring that the controller designed in this paper is meaningful. Figure 11 depicts the change curve of the control input ν of the system. The EMATs system remains stable with the change of environment temperature T and lift-off distance h, and the impedance angle is finally stabilized in a certain set, which concludes that the system works near the resonant frequency.

To further demonstrate the resonant frequency tracking control effect under the proposed control method, we compare the tracking error norm and the norm of the tracking error derivative with the terminal sliding mode control (TSM) tracking strategy. As shown in Figure 12, the dotted line is the norm of the resonant frequency tracking error and error derivative under the TSM strategy, and the solid line is the norm of the resonant frequency tracking error and error derivative under the FT-NITSM designed in this paper. The error of the FT-NITSM tracking control strategy can quickly converge to near the steady state within 5 s, and the final change amplitude is less than 0.05, while the TSM tracking control strategy changes more than 0.4 when it tends to be stable. Comparison results further demonstrate the effectiveness and efficiency of the control strategy designed in this paper.

## 5. Conclusions

In summary, this paper proposes a nonsingular integral terminal tracking control strategy with fixed time strategy (FT-NITSM) for resonant frequency tracking of EMATs considering working environment changes. Simulation results show that the performance of the fixed time convergence control algorithm designed in this paper is superior to the traditional linear control algorithm and the finite time convergence control algorithm. Moreover, the FT-NITSM control strategy can ensure that the operating frequency of the system can accurately track the natural resonant frequency within 3 s, and with the positive and negative tracking error is less than $0.01\times 10^{4}$ Hz. The tracking effect is improved compared with the current resonant frequency tracking control methods. At the same time, ensure that the overshoot range of the system impedance angle change is between −20 and 20, and the convergence speed is fast. The impedance angle maintains a steady state at about 30 s, and the change range is stable between $-5\;\mathrm{and}\;5^{{}^{\circ}}$. Finally, the effectiveness of the FT-NITSM control strategy is demonstrated through error norm comparison experiments. In the future work, to increase the reliability of the conclusions, a specific EMATs resonant frequency tracking experimental platform will be built based on theoretical research. Moreover, the EMATs mathematical model can be further optimized, especially on its robustness and reliability. Finally, to further improve the resonant frequency tracking control effect, the influence of internal and external disturbances of EMATs system on the controller performance should be solved when designing the controller with fixed time control and sliding mode control.

## Figures and Tables

**Table 1:** Parameters and values applied in the simulation.

Parameters	Values	Parameters	Values	Parameters	Values
$a_{1}$ $,a_{2}$	1	$a_{3}$ $,a_{4}$	1	$b_{1}$	3
$b_{2}$	0.33	$b_{3}$	1.5	$b_{4}$	0.5
$\lambda_{0}$	0.2	$\lambda_{1}$	1.0	$\lambda_{2}$	0.9
m	7	n	9		

## Data Availability

The data used to support the findings of this study are available from the corresponding author upon request.
